# Folate Augmentation of Treatment – Evaluation for Depression (FolATED): protocol of a randomised controlled trial

**DOI:** 10.1186/1471-244X-7-65

**Published:** 2007-11-15

**Authors:** Seren Haf Roberts, Emma Bedson, Dyfrig Hughes, Keith Lloyd, Stuart Moat, Munir Pirmohamed, Gary Slegg, Richard Tranter, Rhiannon Whitaker, Clare Wilkinson, Ian Russell

**Affiliations:** 1 North Wales Section of Psychological Medicine, Institute of Medical and Social Care Research (IMSCaR), Bangor University, Academic Unit, Wrexham Technology Park, Wrexham, LL13 7YP, UK; 2 Centre for Economics and Policy in Health, IMSCaR, Bangor University, Dean Street, Bangor, Gwynedd, LL57 1UT, UK; 3 Psychological Medicine, Swansea University, Clinical School, Room 213, Grove Building, Swansea, SA2 8PP, UK; 4 Department of Medical Biochemistry and Immunology, University Hospital of Wales, Health Park, Cardiff, CF14 4XW, UK; 5 Department of Pharmacology and Therapeutics, University of Liverpool, Ashton Street, Liverpool, L69 3GE, UK; 6 North West Wales NHS Trust, Ysbyty Gwynedd, Bangor, Gwynedd, LL57 2PW, UK; 7 North Wales Organisation for Randomised Trials in Health (NWORTH), Institute of Medical and Social Care Research (IMSCaR), Bangor University, Ardudwy, Normal Site, Bangor, Gwynedd, LL57 2AS, UK; 8 Department of General Practice, Cardiff University, Gwenfro Building, Wrexham Technology Park, Wrexham, LL13 7YP, UK; 9 Institute of Medical and Social Care Research (IMSCaR), Bangor University, Brigantia Building, Bangor, Gwynedd, LL57 2AS, UK

## Abstract

**Background:**

Clinical depression is common, debilitating and treatable; one in four people experience it during their lives. The majority of sufferers are treated in primary care and only half respond well to active treatment. Evidence suggests that folate may be a useful adjunct to antidepressant treatment: 1) patients with depression often have a functional folate deficiency; 2) the severity of such deficiency, indicated by elevated homocysteine, correlates with depression severity, 3) low folate is associated with poor antidepressant response, and 4) folate is required for the synthesis of neurotransmitters implicated in the pathogenesis and treatment of depression.

**Methods/Design:**

The primary objective of this trial is to estimate the effect of folate augmentation in new or continuing treatment of depressive disorder in primary and secondary care. Secondary objectives are to evaluate the cost-effectiveness of folate augmentation of antidepressant treatment, investigate how the response to antidepressant treatment depends on genetic polymorphisms relevant to folate metabolism and antidepressant response, and explore whether baseline folate status can predict response to antidepressant treatment.

Seven hundred and thirty patients will be recruited from North East Wales, North West Wales and Swansea. Patients with moderate to severe depression will be referred to the trial by their GP or Psychiatrist. If patients consent they will be assessed for eligibility and baseline measures will be undertaken.

Blood samples will be taken to exclude patients with folate and B12 deficiency. Some of the blood taken will be used to measure homocysteine levels and for genetic analysis (with additional consent). Eligible participants will be randomised to receive 5 mg of folic acid or placebo. Patients with B12 deficiency or folate deficiency will be given appropriate treatment and will be monitored in the 'comprehensive cohort study'. Assessments will be at screening, randomisation and 3 subsequent follow-ups.

**Discussion:**

If folic acid is shown to improve the efficacy of antidepressants, then it will provide a safe, simple and cheap way of improving the treatment of depression in primary and secondary care.

**Trial registration:**

Current controlled trials ISRCTN37558856

## Background

Clinical depression is common, debilitating and treatable; one in four people experience it during their lives. By 2020, unipolar major depression is predicted to be the second leading cause of disability worldwide [1]. Impaired physical, social and occupational functioning are characteristic of depression, as is increased mortality via suicide, alcohol and drug misuse, and increased rates of cardiovascular disease [2]. Depression thus burdens individuals, families, the NHS, and the national economy [3]. Sub-optimal treatment of depressive disorders is therefore of great public health concern. Mental health is, like cardiovascular disease, the subject of a National Service Framework and thus reflects the priority given to the recognition and management of depression.

Despite a striking increase in the number of antidepressant options over the last 50 years their effectiveness remains largely unchanged. In line with National Institute of Clinical Excellence (NICE) guidance [4] the great majority of recognised sufferers are treated in primary care. However only half respond well to active treatment, while one-third respond to placebo [5]. According to NICE guidelines [4] selective serotonin reuptake inhibitors (SSRIs) are as effective in outpatient depression as tricyclic antidepressants (TCAs); since SSRIs generally have fewer side effects, they are recommended as first-line treatment in primary care.

The monoamine hypothesis of depression implicates a functional deficiency of noradrenaline (NA) or serotonin (5-hydroxytryptamine, 5-HT) in neurotransmission; virtually all antidepressants are thought to act by prolonging the activity of these neurotransmitters or by modulating receptor sensitivity [6]. Folate is an essential cofactor for the biosynthesis of both 5-HT and NA. Thus folate deficiency leads to impaired 5-HT synthesis in the human brain [7]. Moreover, studies demonstrate that up to one-third of patients with depressive illness have decreased plasma and red cell folate levels [8]. This may result from poor nutrition or socio-economic disadvantage, both common in chronic mental illness. Patients with low folate respond less well to antidepressant therapy [9]. However, current clinical measures of folate status do not detect patients who may have functional rather than absolute deficiency.

Homocysteine, a toxic amino-acid metabolite elevated in functional folate deficiency, is a highly sensitive marker of folate status. A recent cohort study demonstrated that hyperhomocysteinaemia (plasma level >15 μmol/L), but not total serum folate or vitamin B12, is significantly related to depression severity (odds ratio = 1.90; 95% confidence interval = 1.11–3.25)[10]. Another study that examined 412 people aged between 60 and 64 years found that low folate and high homocysteine, but not low vitamin B12 levels, are correlated with depressive symptoms [11]. Further evidence of a possible role of impaired folate metabolism in depression is suggested by a finding that patients homozygous for an abnormal variant of the methylenetetrahydrofolate reductase gene experience more severe depression (odds ratio = 1.69; 95% confidence interval = 1.09–2.62)[12]. This study has not been replicated, however, and was associated with a relatively modest odds ratio, less than expected with homocysteine. The use of genotyping to predict the effectiveness of folate supplementation of antidepressants thus needs further critical examination in appropriately powered studies that also take into account functional measures such as homocysteine level. Such a strategy should also acknowledge that folate metabolism in the human body is extraordinarily complex; 27 enzymes are involved, many of which exhibit polymorphisms [13].

A variety of evidence thus suggests that folate may be a useful adjunct to antidepressant treatment: 1) patients with depression often have a functional folate deficiency; 2) the severity of such deficiency, indicated by elevated homocysteine, correlates with depression severity, 3) low folate is associated with poor antidepressant response, and 4) folate is required for the synthesis of neurotransmitters implicated in the pathogenesis and treatment of depression. Despite such suggestive evidence few attempts have been made to determine the effect of folic acid supplementation on drug treatment of depression. A Cochrane systematic review and meta-analysis identified only two randomised controlled trials (combined n = 151) examining the role of folate augmentation in depression [14,15]. The trials differed substantially in recruitment criteria and provide little evidence for or against the routine use of folic acid in antidepressant treatment. In only one of the studies was folate given in its dietary form, in this case combined with fluoxetine [16]. Folic acid at only 0.5 mg per day was adequate to change serum homocysteine status, and to enhance antidepressant action, in females but not males. Thus the differential effect in men and women may have been due to the low dose of folic acid used in the study. In addition, abnormal clinical chemistry, (e.g. raised mean corpuscular volume as seen in folate deficiency) was used to exclude patients. The other study utilised a higher dose of methylfolate (15 mg) and selected patients with low red cell folate (<200 μg/L) but was of low power, with only 24 patients [17]. No study to date has used pharmacological doses of folic acid (i.e., 5 mg daily) to augment pragmatic antidepressant therapy. Hence there is a clear need for a trial with sufficient power to examine the effect of folic acid supplementation in the treatment of clinical depression. The effects of folic acid supplementation may vary across different clinical populations encountered in primary and secondary care; in particular, the clinical and cost effectiveness of folate augmentation for new cases may differ from that of cases with continuing but ineffective antidepressant prescription. In addition, the biological mechanism of folate augmentation may differ across different antidepressant treatments; accordingly, a comparison of SSRI versus other antidepressants is of interest.

The evidence, reviewed above, suggests that folic acid supplementation may augment antidepressant response. This is consistent with the finding that baseline levels of folate within the normal range predict antidepressant response [18]. In addition, there is evidence to suggest that folate deficiency, (e.g., as may be produced by inflammatory disorders or anticonvulsant drug treatment [19]), may aggravate depression or impair response to treatment.

Since dose-response relationship for folic acid as an adjunct to antidepressant treatment is not known, it is necessary to consider other evidence with regard to appropriate dose choices for use with antidepressant. Based on known pharmacology of folate, including its penetration into the central nervous system and effects on neurotransmitter metabolism, there is little chance that 5 mg would be *less *effective than lower dose (e.g., 0.5 mg/day) folic acid, whereas it could well be *more *effective. This argument is bolstered by findings [20,21] that 5 mg/day is more effective than 0.4 mg/day with respect to both clinical and pre-clinical outcomes in the human cardiovascular system. There is little available information regarding the mechanism that may underlie a possible antidepressant-adjuvant effect, although suppression of homocysteine (Hcy) and clinical improvement were both more common in women than men treated with fluoxetine combined with folic acid [16]. This result suggests that higher doses of folic acid may be more effective, especially in men. Further evidence regarding potential mechanisms may be inferred from cardiovascular data. In this system there is growing evidence that Hcy elevation is a marker of functional folate deficiency, but suppression of Hcy by folic acid does **not **correlate with the latter's beneficial effects. For example, 0.4 mg/day folic acid is adequate to suppress elevated Hcy, but not to restore disturbed endothelial function measured using the technique of flow mediated dilatation of human forearm artery [22].

Furthermore, despite high doses (5 mg) of folic acid being substantially above the FDA's recommended daily dose (0.4 mg), there are few risks associated with such high doses other than for those with B12 deficiency, epilepsy or malignant disease. Even with lower doses of folic acid, consultant haematologists advise that vitamin B12 screening, and exclusion of those found deficient, would be required. With the exception of patients with malignant disease and some patients with epilepsy, high dose folate is known to be safe in various clinical populations, including pregnant women and the elderly, provided B12 deficiency is screened and excluded [23]. Thus providing those patients with contraindicated conditions or treatments are excluded there is no experimental or clinical disadvantage to using 5 mg/day folic acid dose.

Thus consideration of the appropriate folic acid dose for trial participants with normal baseline serum folate is limited by the lack of prior trials of folate augmentation of antidepressant treatment with essentially no information available on the nature of the dose-response relationship apart from the observation that women appear to respond better than men to low dose (0.5 mg/day) folic acid augmentation of fluoxetine [16]. Accordingly, the decision regarding folic acid dose depends on considerations of safety and possible effectiveness of various doses, ranging from 0.4 mg/day (as is used routinely pre-conception and in early pregnancy) to 5 mg/day (as is used routinely to treat folate deficiency). Of the two available tablet doses, we chose a pharmacological dose of 5 mg/day to improve our chances of detecting an effect of folate supplementation in depression.

## Methods/Design

The primary objective of this trial is to estimate the effect of folate augmentation in new or continuing treatment of depressive disorder in primary and secondary care. Secondary objectives are to evaluate the cost-effectiveness of folate augmentation of antidepressant treatment, investigate how the response to antidepressant treatment depends on genetic polymorphisms relevant to folate metabolism and antidepressant response, and explore whether baseline folate status can predict response to antidepressant treatment.

**Figure 1:**
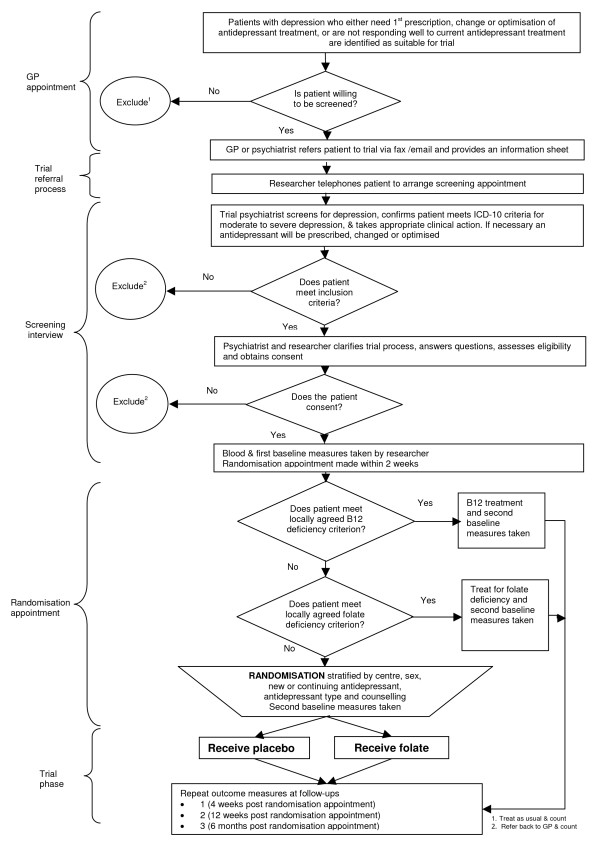
Flow diagram of trial.

This is a multi-centred double blind, placebo-controlled, randomised trial of folic acid augmentation of pragmatic antidepressant treatment of moderate-to-severe depression. The trial investigates the effect of folic acid augmentation on new and continuing antidepressant treatment over 3 months. Assessments will be at week -2 (baseline 1 – antidepressant initiation if required), week -1 (telephone contact for tolerability of antidepressant), week 0 (baseline 2 – randomisation to folate or placebo), and weeks 4, 12 and month 6 (outcome measures). Figure 1 shows the flow diagram of the trial.

### Outcome measures

To estimate the effectiveness of folic acid in augmenting antidepressant treatment the trial will measure changes in depressive symptoms both from the participant's own experience and from the clinical perspective. The primary outcome measure is symptom severity as estimated by the self-rated Beck Depression Inventory (BDI). Clinician-rated Montgomery-Asberg Depression Rating Scale (MADRS) and the clinical global impression (CGI) of change will also measure symptom severity as secondary measures. Secondary outcome measures will include: health status (mental and physical aspects of quality of life) through the SF-12; recording and appropriately reporting of adverse events (e.g. psychiatric inpatient admission, attempted or completed suicide, and other mortality); and side effects as measured by the UKU side effects scale [24]. Cost-utility analysis will use the EuroQoL (EQ-5D), a self complete resource use instrument and relevant medication history from GPs.

Blood samples at baseline and each follow-up will measure folate and B12 status. Homocysteine levels will be determined from blood samples taken at baseline, week 12 and month 6. Pharmacogenetic analysis will be carried out on blood samples obtained at baseline only.

Compliance will be measured using the following four methods:

(1) The number of tablets remaining at each follow up will give a crude estimate of maximum compliance.

(2) Dispensing records for folic acid (or placebo) will be reviewed to measure persistence with therapy; and dates on antidepressant medication containers to indicate delay in collecting repeat prescriptions.

(3) Patients will be asked to complete the Morisky questionnaire [25], a validated tool for assessing compliance with tricyclic antidepressants [26] at the 12-week visit only.

(4) Red cell folate and homocysteine levels will be used as biochemical measures of compliance.

### Referral and recruitment

Patients will be recruited from primary and secondary care in three NHS Trusts areas – North East Wales, North West Wales and Swansea, covering a population of about 800,000. A feasibility study in North West Wales established two ways of improving the recruitment of patients from primary care into antidepressant trials: (a) researchers visited general practices to register, consent, randomise and follow-up patients; and (b) responsibility for prescribing shifted from general practitioners to trial psychiatrists. As a result the study recruited 155 patients in 18 months at a single centre with fewer resources than proposed for FolATED. Where appropriate this study will adopt a similar model in order to reach our recruitment target. In addition to minimising the barriers to recruitment we have developed two further strategies. First, GPs will be reimbursed for the administration costs for each patient recruited into the trial and second, patients will complete a depression rating scales (BDI, HADS or PHQ9) in accordance with GP preferences. These strategies will ensure the proposed sample size is achievable.

Research interested general practices will be identified in each of the three Trust areas, from the All-Wales Primary Care Research Database managed by the North Wales Department of General Practice. Inviting general practices with a proven interest in research to refer patients to the study can be an effective way of enhancing recruitment rates. The feasibility study in North West Wales demonstrated that research interested practices can recruit up to 20 patients each over a 2-year recruitment period. Thus 365 patients will be recruited from primary care.

The remaining 365 patients will be recruited from secondary care. Although most cases of depression are managed in primary care across Wales, additional diagnostic and management support is available in somewhat different configurations in the three target regions. At North East Wales NHS Trust, patients with common mental health problems unable to be managed by the GP are referred to the First Access Mental Health Service; by contrast only patients with severe and enduring mental illness are referred to the Community Mental Health Teams (CMHTs). At North West Wales NHS Trust, patients with depression requiring referral are directed either to the practice counsellor, to research projects (e.g. the feasibility study previously mentioned), or to the local CMHT. Similarly, at Swansea NHS Trust patients with depression not managed effectively in primary care are seen by liaison psychiatric nurses and may be referred to the Psychiatrist within the local CMHT.

The Consultant Psychiatrists at each centre will ensure that their respective teams recruit 122 patients over the 18-month recruitment period, giving a total of 366 patients recruited from secondary care. It is anticipated that patients recruited from secondary care will predominantly be continuing cases that have already been initiated on antidepressant treatment. To maximise recruitment the study will be adopted by the Mental Health Research Network Cymru (MHRN-Cymru).

Participating GPs will identify patients with depression and will give them a study booklet if they are interested in entering the trial. The study booklet will contain details of the trial in the patient information leaflet and consent form. Patients willing to be screened will be referred to the trial using a standardised referral form which will be faxed to the regional research team. In addition to the patients' demographic details, the form will also ask the GPs to note their first and second choice antidepressant in each instance if they have not been prescribed an antidepressant. Patients not responding adequately to existing antidepressant treatment will also be referred to the trial in a similar manner. Patients with moderate or severe depression routinely referred to secondary care will also be recruited into the trial using the same referral process. The local research professional will contact referred patients within three days to discuss the study, answer any questions and to arrange an appointment for a screening interview at the GP surgery or local research centre. Patients who do not wish to be screened or are ineligible for other reasons will be offered usual treatment by the GP. To avoid withholding treatment an appointment will be made within three working days for new patients who have yet to be prescribed an antidepressant. For patients already prescribed an antidepressant an appointment will be made as soon as possible.

### Screening

At the screening interview the Psychiatrist explains what will happen and will ask the patient if they are happy to be assessed. Patients will be assessed for depression by the psychiatrist and asked to complete the BDI as part of the screening process. (If the GP requests HADS or PHQ9 patients will be asked to complete them in addition to the BDI.)

The Psychiatrist will take an appropriate clinical action based on the patients needs such as prescribing or optimising an antidepressant, or onward referral. If the patient meets ICD-10 criteria for moderate to severe depression the Psychiatrist will ask the patient if they are willing to be screened for eligibility. See planned inclusion and exclusion criteria.

### Inclusion and exclusion criteria

#### Inclusion

Only patients aged 18 or over with an ICD-10 diagnosis of moderate to severe depression [confirmed by the trial psychiatrists during the screening interview using BDI] will be included. Only patients able to give informed consent (not delirious, actively psychotic or with severe communication or learning disability) and able to complete the research assessments will be included.

#### Exclusion

Patients will be excluded from the trial if they:

(a) are folate deficient: they cannot be randomised because they need to be treated with folic acid but can be included in the comprehensive cohort

(b) are B12 deficient: they cannot be randomised because they need to be treated with B12 injections but can be included in the comprehensive cohort

(c) have knowingly taken supplements containing folic acid within 2 months because this will mask any effects of folic acid given during the study

(d) suffer from psychosis because additional treatment for psychosis may mask any benefit of folic acid with antidepressants. Plus people suffering from psychosis are less able to give informed consent and will require referral through to secondary services.

(e) are already participating in another research project

(f) are pregnant or planning to become pregnant as it is important for pregnant women to take folic acid so they cannot be randomised to placebo

(g) are taking anticonvulsants as in very rare circumstances folic acid can react with certain anticonvulsants

(h) serious, advanced or terminal illness with a life expectancy of less than 1 year

(i) have recently started treatment for a medical condition which has not yet been stabilised

(j) are taking lithium

(k) have had a diagnosis or treatment for any malignant disease or any related condition such as intestinal polyposis

### Informed consent

Once eligibility is determined the research professional and psychiatrist will provide information about the trial and the patient will be given the opportunity to ask any question regarding the trial. The patient is told that they can withdraw from the study at any time without their usual care being affected. The research team will check that the patient understands all aspects of the trial including the genetics part. The psychiatrist must be happy that the patient is willing to consent to the trial. Patients will then be asked to give informed consent to participate in the trial. If the patient agrees to enter the trial, the patient must complete two copies of the consent form indicating which parts of the study they are consenting to. Once the patient has completed the consent form the Psychiatrist must sign and date it. One copy of the study booklet with completed consent form will be given to the patient and one copy will be kept by the research team. Patients who do not wish to enter the trial will be referred back to their GPs.

### Baseline measures and blood samples

First baseline measures will be undertaken during this screening appointment. For new cases of depression, the GPs can initiate according to their usual practice or they can refer the patients to the study and the trial psychiatrists can initiate treatment. To be pragmatic, prescriptions will take account of preferences expressed by GPs. Where the GP does not state a preference one of two available generic SSRIs, namely citalopram or fluoxetine, will be prescribed. SSRIs will be prescribed according to NICE recommendations. For depression not responding adequately to treatment, the trial psychiatrist will continue with the current prescription where appropriate and adjust dosage in accordance with the current British National Formulary [27]. If optimisation is required, patients will be given an optimal dose for 4 weeks before randomisation.

Note that two key exclusion criteria, namely B12 or folate deficiency, will be determined via a blood test. For this reason, blood samples will be taken at the screening interview to determine eligibility in the first instance.

Blood samples (40 ml in total) will be split into three, collected in EDTA tubes (plus a serum gel tube for haematology):

(1) Routine haematology. Full blood count, red cell folate, serum folate and B12, to be analysed in local NHS laboratories in Bangor, Wrexham and Swansea.

(2) Homocysteine. Samples will be centrifuged within 1 hour and plasma taken off red cells. The resulting plasma will be stored at -20°C and sent batch-wise to the University Hospital of Wales, Cardiff.

(3) Genetic analysis (if consent is given). Samples will be sent, as and when taken, to the University of Liverpool.

A further research appointment will be arranged between the research professional and the patient within 14 days, in which time the folate and B12 results will be confirmed. A copy of the results will be sent to the patient's GP, again using a standardised form.

Patients who are ineligible or who do not consent to participate in the trial will be referred back to their GP for usual care. When needed an antidepressant will be prescribed to avoid further delays in treatment and any appropriate clinical action will be taken. A standardised form will be used for referrals back to GPs.

### Blood results and randomisation

Upon receipt of the blood results, the research professionals will liaise with the trial psychiatrist to determine B12 and folate status based on locally determined values. The psychiatrist will then prescribe the appropriate action to be taken at the second appointment between the research professional and the participant. At this appointment the research professionals will inform the participant of their blood test results and their eligibility to continue in the trial. Patients whose blood results show B12 deficiency will be referred back to their GP for immediate treatment with B12 injections. GPs will be informed of the deficiency with a copy of the blood results. Those patients will be excluded from the principal trial but will continue in the 'comprehensive cohort study' of recruited patients. Thus antidepressant response will be monitored at each follow-up.

Patients whose serum folate results suggest folate deficiency will have their red cell folate levels reviewed to confirm the deficiency. If red cell folate is also below the normal range for the local laboratory, then we shall exclude the participant from the trial, but include him or her in the 'comprehensive cohort study' for follow-up during (unblinded) folate supplementation. We shall then inform the GPs of the required treatment with copies of the blood results and continue to monitor treatment responses at each follow-up.

### Randomisation, stratification and blinding

Following baseline observations, patients with B12 and folate levels within the normal range will be randomised and allocated to folic acid or matching placebo. Participants will thus receive either a folic acid or placebo adjunct to their antidepressant treatment. Randomisation to FoLATED will be achieved by telephone to the remote randomisation centre at North Wales Organisation for Randomised Trials in Health (NWORTH) at Bangor University. The randomisation will be performed by dynamic allocation to protect against subversion while ensuring that each arm of the trial is balanced for the stratification variables. Participants will be stratified by (1) centre (Swansea/Wrexham/Bangor); (2) sex (male/female); (3) patient type [new/continuing (i.e. having taken the same daily antidepressant for at least two months with a stable dose in the therapeutic range (BNF) for at least one month)] (4) the type of antidepressant prescribed (SSRI/other) and (5) whether or not they have ever received counselling for depression.

For validation purposes, additional information is also requested including the participant's trial number, date of birth, and the name of the person requesting the randomisation. The following questions will be asked during the randomisation process:

1. Has consent been given?

2. Does the patient meet ICD10 criteria?

3. Is the patient B12 deficient?

4. Is the patient folate deficient?

If a person requesting the randomisation responds 'Yes' to the first two questions and then 'No' to the second two questions the participant can be randomised.

Identically packaged folic acid and placebo will be coded randomly for each stratification group by NWORTH. Both folic acid and placebo tablets will appear identical. In this way the patient, doctor, researchers and pharmacists will be blind to the intervention. The research professionals at each centre will hold the trial drugs and distribute as necessary. Each patient's prescription will indicate his or her trial number and package serial number in addition to the randomisation code generated by NWORTH. This will determine the appropriate trial package to be dispensed. The allocated codes will be recorded in the research notes and the patient's clinical record. The key to the randomisation code will be held centrally by NWORTH and by the local pharmacies. A telephone number of the local pharmacy will also be available so that the code can be broken in an emergency.

### Follow-Ups

There are clinical and methodological reasons for following up after 4 weeks, 12 weeks and 6 months. Evidence suggests that all antidepressants show a delayed and variable onset of clinical improvements in depression [28-30]. Although some antidepressants reportedly produce significant improvements within the first week, this typically continues over subsequent weeks; variability in response measures also affects the reported timing of onset of response [28,29]. Available evidence indicates that around half of eventual responders (judged in week 8) to fluoxetine treatment started to respond within two weeks and that 75% started to respond within four weeks. A lack of response within four or six weeks was associated with 75% or 88% chance respectively of non-response within eight weeks. However no predictors of response timing were found. Thus the onset of antidepressant response, if any, will most likely have occurred by week 6. We therefore propose to undertake the first outcome measures follow-up at week 4 (6 weeks after initiation of antidepressant treatment, or optimisation for continuing patients) to ensure sufficient time for likely treatment effects whilst retaining sensitivity to clinical change. In addition non-response at this point allows for possible changes to the antidepressant treatment in accordance with the British National Formulary and NICE guidelines as stipulated below. Given this early follow-up, we propose to undertake later follow-up at 12 weeks to measure late and continued responses to antidepressant treatment. It is important to monitor relapse rates during the maintenance phase of treatment and to identify any effects of folate on late responders to antidepressants. Evidence suggests that SSRIs are associated with higher rates of relapse (tachyphylaxis) than other antidepressant classes including tricyclic antidepressants (TCAs) and selective noradrenaline reuptake inhibitors (SNRIs) [31]. Thus follow-up at 12 weeks monitors, not only the effects of folic acid on clinical outcomes, but also its effect on relapse rates.

For new patients or continuing patients undertaking optimisation, additional telephone follow-ups in week -1 will gauge tolerability of treatment and suicidality. Where necessary, changes to antidepressant type or dose will be made. These changes will be the subject of regular summary reports to the trial Data Monitoring and Ethics Committee (DMEC).

There are two methodological reasons for follow-up after 4 weeks, 12 weeks and 6 months. First, one of these is during treatment with folate, one and the end of treatment and the final one after some time has lapsed post treatment. Second, the combination of short-, medium- and long-term measures yields better estimates of the cost-effectiveness of treatment.

With consent, serum folate, homocysteine and B12 levels will be assessed at week 12 and at the final follow-up. A summary of the baseline and follow-up assessments can be seen in Table 1.

**Table 1:** FolATED Baseline and follow-up assessments

**Assessment**	**Outcome measure**	**Respondent**	**Trial entry – Baseline**			**Follow-ups**		
			
			Week -2 (baseline 1)	Week -1 (Telephone contact)	Week 0 (baseline 2)	Week 4	Week 12	Month 6
Blood testing	Routine haematology							
	FBC	Clinician						
	Serum folate	Clinician						
	Red Cell folate	Clinician						
	B12	Clinician						
	Homocysteine	Clinician						
	Genetics	Clinician	(needs extra consent)					
Depression status	BDI	Patient						
	MADRS	Clinician/researcher						
Health status and quality of life	CGI	Clinician/researcher						
	SF-12	Patient						
	EQ-5D	Patient						
Health economics	Resource usage	Patient						
Compliance and side effects	Morisky Questionnaire	Patient						
	UKUside effects scale	Clinician/researcher						

### Withdrawal

A participant can withdraw or can be withdrawn from the treatment. Participants who withdraw from the trial treatment will be asked to attend all the follow-up appointments. Local research co-ordinators will notify the trial co-ordinator in writing about all trial participants who wish to withdraw from all future follow-ups.

### Pharmacogenetics Study

There is increasing realisation of the potential importance of pharmacogenetics in maximising the benefits of medicines and to this end, most commercial trials and an increasing number of non-commercial trials, now contain a pharmacogenetic arm. For this reason, a major strand of this trial is to characterise the role of genetically determined variation in folate metabolism in clinical outcome.

Prior to the completion of the human genome project, the conventional strategy utilised for pharmacogenetics involved the analysis of single variants in single genes. This has been practiced since the 1960s and unfortunately has not led to major breakthroughs. Indeed the literature is littered with contradictory data on pharmacogenetics, which has made it impossible to translate any benefits into clinical practice. As stated earlier, folate metabolism is complex involving 27 different enzymes [13]. The complexity is exacerbated by the fact that folate requires active transport into cells, and the facilitated transporters show genetic polymorphisms [32]. To date, only the common functional genetic polymorphism (C677T) in methylenetetrahydrofolate reductase (MTHFR) gene has been related to depression [12], although no study has yet related the occurrence of this polymorphism to the effectiveness of folate treatment. No other genes in the folate pathway have been investigated either in relation to the severity of depression, response to antidepressant treatment, or to folate supplementation.

Concentrating on just one gene (for example MTHFR, the most widely studied gene in the folate pathway) would be unnecessarily restrictive and would ignore the variance in the other pathways. On the other hand, in the timescale available in this study, it would not be possible to undertake an analysis of all 27 genes involved in folate metabolism because (a) the genetic variability has not been adequately characterised, (b) the functional effects of all genetic polymorphisms has not been investigated, and (c) the study has not been powered to look at this number of genes with an insufficient sample size.

Furthermore, this would also be prohibitively expensive at this stage. A practical strategy has been adopted, as follows:

(a) analysis of the most important pathways based on previous studies in other diseases areas, for example response to methotrexate;

(b) we will adopt a genetic strategy that involves using tagging SNPs to determine the overall genetic diversity of the chosen genes. The tagging SNPs will be determined from the data that are publicly available via the HapMap project;

(c) we will genotype patient samples for the common polymorphisms (>10% population frequency) that have either a known functional effect or can be predicted to have a functional effect and would therefore be in accordance with the sample size and hence power of the study; and

(d) DNA will be stored for future analysis for other variants in the same and other genes.

This strategy is currently being tested by Prof. Pirmohamed as part of 3 large projects funded through the Department of Health Pharmacogenetics Initiative. This strategy will provide the highest chance of identifying genetic predictors of responsiveness to folate whilst simultaneously provide us with a resource, as knowledge of the genetics of these pathways increases, for future refinement and identification of other associations.

Thus, although this is a feasible strategy, it will nevertheless be the most thorough investigation to date by allowing us to determine critically whether the interaction between any of the genetic polymorphisms in the folate pathway predicts the severity of depression, response to antidepressants *per se*, and the response to folate supplementation. Furthermore, this will allow us to determine whether such a strategy has an impact on patient outcome, and therefore whether it is clinically and cost-effective in a real-world setting. With this in mind, the following 7 (out of the possible 27) genes in the folate pathway will be investigated in the first instance:

• methylenetetrahydrofolate reductase

• thymidylate synthase

• dihydrofolate reductase

• methionine synthase

• methionine synthase reductase

• gamma glutamyl hydrolase

• reduced folate carrier (SLC19A1).

The choice of genetic variant within each gene will be based on (a) literature review of the functionally important common polymorphisms; (b) a search of the publicly available databases including dbSNP ; and (c) a search of the HapMap database.

DNA will be extracted according to standard procedure. Genotyping for polymorphisms will be undertaken using a medium throughput platform utilising TaqMan technology – available in the Department of Pharmacology, University of Liverpool [33]. It is difficult to provide realistic power calculations in advance since little is known about the frequency of genetic variants in patients with depression and the statistical methodology for the analysis of genetic association studies is still being developed. A key variable in such studies is the frequency (among cases) of the SNP minor allele frequency. For rarer variants to be clinically important, their effect size (measured by the allelic odds ratio, OR) must be large. We therefore specify distinct benchmarks for the power analyses: we seek to have good power for (a) OR = 3 and a variant with a frequency of 10%; (b) OR = 2 for a common variant (>20%). Note that these effect sizes are for a single causal variant; we expect to realise much larger effect sizes via combinations of causal variants. The assumed type 1 error is 5%. Adjustment for multiple testing will be performed by permutation analysis [34]. As indicated above, multiple regression models will be used to evaluate the importance of genetic factors in determining clinical outcomes in patients.

### Intervention

Given the increased efficacy of higher folic acid doses in other body systems and the very low risk of adverse effects, we have opted for a pharmacological 5 mg/day folic acid dose or matching placebo to supplement antidepressant treatment, the same dose as is routinely used to treat folate deficiency. This dose is the treatment of choice for folate deficiency and well tolerated in various clinical populations, including the elderly and pregnant women [35,36]. There is no evidence that pharmacological folate will have a deleterious effect on either methylation status or mood, nor that it will lead to sub-acute combined degeneration of the spinal cord or other neurological complications, provided that B12 deficiency is excluded. Individuals identified with B12 or folate deficiency will be excluded from the folate/placebo trial, but will be treated appropriate and asked to continue to participate in the 'comprehensive cohort study'. People who have been diagnosed or treated for malignancy will not be able to receive folic acid. Although there is evidence that high folate intake lowers the risk of developing cancer if a person has already had a malignant disease there is some evidence that high folate intake may increase cancer growth. In summary,

• 5 mg/day folic acid is safe in working age adults and the elderly, provided B12 deficiency and diagnosed or treated malignancy is excluded.

• 5 mg/day folic acid is more likely than lower doses to be effective as an antidepressant adjuvant, based on limited trial data and folate's known effects in other body systems.

Additional advantages include simplicity, low cost and ready availability of the 5 mg preparation [27]. The folic acid dose-response relationship in clinical populations, in particular those with depression, will need to be determined in subsequent studies. Patients with folate deficiency continuing in the comprehensive cohort study will be treated with a 5 mg dose of folic acid. Patients with a B12 deficiency also continuing in the comprehensive cohort study, will be treated with B12 injections as necessary by the GP. Patients not wishing to continue will be referred back to their GP for appropriate treatment using the designated form.

### Risks and anticipated benefits for trial participants and society, including how the benefits justify the risks

There is suggestive evidence that folate may enhance the effects of antidepressants for those suffering depression. Thus patients requiring antidepressant treatment may benefit from supplementing their treatment with folic acid. The associated risks with folic acid supplementation are very low. Folic acid is normally well tolerated but occasionally nausea, allergic reactions, anorexia and abdominal distension can occur. However, there is a potential risk for patients with B12, folate deficiency or malignancy. Those patients with B12 deficiency must not receive folate and will be excluded from the trial. They will be asked to continue to participate as part of the 'comprehensive cohort study' and will be referred back to their GP for immediate treatment with B12 injections. Similarly, patients with folate deficiency must be treated with folic acid and it would be unethical to randomise these patients to receive placebo. For this reason, they will also be invited to continue to participate as part of the comprehensive cohort in an unblinded folate group and monitored as appropriate.

Patients with mental health problems might be viewed as a vulnerable population and therefore appropriate measures will be taken to ensure that they fully understand the nature of the trial and the risks and benefits of the treatment. We recognise that people suffering from moderate to severe depression may have reduced capacity to assimilate information and we will thus ensure that all information is clear, user friendly, honest, and precise. Information will be given verbally in addition to written information sheets and the research professionals will be available to answer any question. If folic acid augmentation is shown to be beneficial, it would be a cheap, safe and simple method of improving treatment of a common, debilitating illness in primary care.

### Informing potential trial participants of possible benefits and known risks of the intervention (or of no intervention or a placebo)

Information sheets will be given to patients to keep which will explain in detail all the benefits and risk of participating in the trial. Research professionals will be available to answer any questions and respond to any difficulty experienced during the trial. Patients will be given the opportunity to nominate an advocate (e.g., family member) if they wish. Participants will be asked not to take folic acid supplements (including multivitamin preparations containing folic acid) outside of the trial and any individual that becomes pregnant during the trial will be asked to inform the appropriate research professional. These patients will be followed-up as intention to treat. At each appointment participants will also be asked if they are taking any additional supplements.

### Safety monitoring and reporting

To ensure that the safety of folic acid is properly monitored and reported this protocol stipulates the definitions used to identify different types of adverse events (AE) and the associated reporting requirements (adapted from the EU Directive [37]). All adverse events will be assessed for causality, seriousness and expectedness. That is whether the AE is related to the drug; whether the AE is serious; and whether the AE was unexpected. Table 2 shows how these three features apply to the main types of AEs.

**Table 2:** Types of adverse events

	**Adverse Events (AE)**	**Adverse Reactions (AR)**	**Serious Adverse Events (SAE)**	**Serious Adverse Reactions (SAR)**	**Suspected Unexpected Serious Adverse Reactions (SUSAR)**
Is the medical occurrence considered to be related to trial drug?	N	Y	N	Y	Y
Is the medical occurrence serious?	N	N	Y	Y	Y
Is the medical occurrence unexpected?	N	N	N	N	Y

### Causality

Causality is the degree to which an untoward medical occurrence can be attributed to the trial drug and can be classed as either unrelated, unlikely to be related, possibly related, probably related or definitely related. Only untoward medical occurrences that are considered to be either possibly, probably or definitely related to the trial drug will be reported as having a causal relationship.

If the untoward medical occurrence *is not *considered to have a causal relationship with the treatment at the time of the event (i.e. it is not believed to be a consequence of taking folic acid or placebo) this will be classified as an Adverse Event. However, if it is considered to have a causal relationship with folic acid or placebo at the time of the event it will be classified as an Adverse Reaction.

### Seriousness

Any untoward medical occurrence will deemed serious if it:

• results in death

• is life-threatening

• requires hospitalisation or prolongation of existing hospitalisation

• results in persistent or significant disability or incapacity

• results in a congenital anomaly or birth defect

Self harm or attempted suicide will also be considered serious in this study.

All serious events not considered to have a causal relationship with the treatment will be classified and reported as a Serious Adverse Event (SAE). All serious events that are considered to have a causal relationship with the treatment will be classified and reported as a Serious Adverse Reaction (SAR).

### Expectedness

An untoward medical occurrence will be considered to be 'unexpected' if its nature and severity are not consistent with the information in the summary of product characteristics for that treatment. If an adverse event is considered 1) to be related to the folic acid or placebo 2) is serious and 3) unexpected then is will be classed as a Suspected Unexpected Serious Adverse Reaction (SUSAR).

Known undesirable effects reported in the summary of product characteristics include nausea, anorexia, abdominal distension and allergic reaction.

### Responsibility for reporting

**Figure 2:**
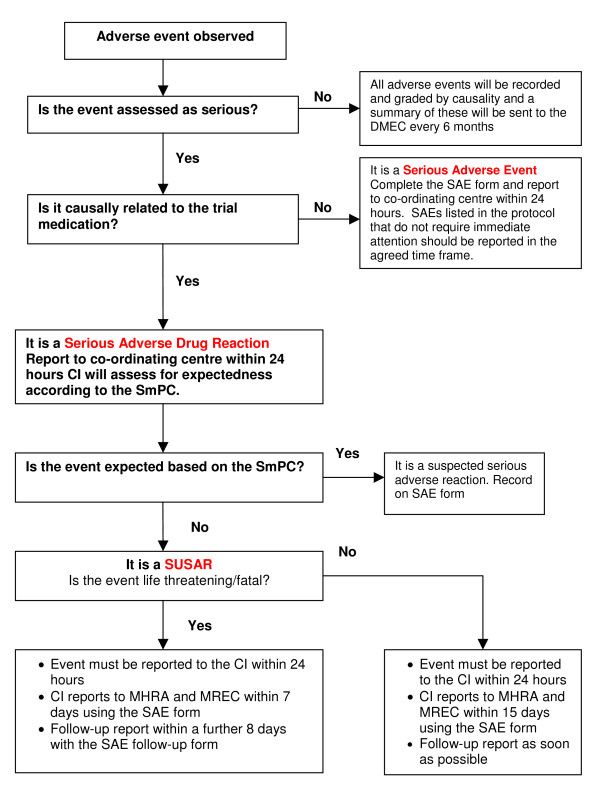
Flow diagram of safety reporting.

The reporting requirements differ depending on the causality, seriousness and expectedness of the medical occurrence as summarised in the flow diagram of safety reporting (Figure 2).

All adverse events will be recorded on the trial database, evaluated by the principal investigator or other designated person responsible for the clinical aspect of the trial in each centre and included in the annual safety report to the MREC and MHRA.

However, for all events considered serious additional reporting is required. The trial co-ordinating centre must be informed within 24 hours of investigator's knowledge of the event. A Serious Adverse Event Form must be completed and sent to the trial co-ordinating centre as soon as possible. The trial co-ordinator will immediately contact the clinical and methodological chief investigators who have been delegated the responsibility of reporting any serious adverse events on behalf of the sponsor (Bangor University). A decision must then be made by the investigator with the clinical responsibility for aspects of the patient's care which are relevant to the trial as to whether an adverse event is related to folic acid or placebo. The investigator should decide the degree to which the event is caused by the trial drug. If a decision cannot be made, the investigator must contact the co-ordinating centre. Advice will be sought from the clinical chief investigator and other clinicians may be asked if a decision cannot be reached.

If it is decided that the adverse event is a serious adverse reaction then it must be determined whether the reaction is expected. If it is unexpected and thus considered a SUSAR then the SAE form will be completed and reported to the MHRA and MREC.

For fatal or life-threatening SUSARs the MHRA and MREC will be notified as soon as possible but no later than 7 calendar days after the sponsor has first knowledge of the event. In each case relevant follow-up information will be sought and a report completed as soon as possible. The follow-up information will be sent to the MHRA and the MREC within an additional eight calendar days.

Non-fatal and non life-threatening SUSARs will be reported to the MHRA and the MREC as soon as possible but no later than 15 calendar days after the sponsor has first knowledge of the event. Further relevant follow-up information will be given as soon as possible.

Serious but expected reactions or non-serious adverse reactions will be recorded in the database and reported in the usual way.

### Statistics

With national collaboration and adoption of the project by the Mental Health Research Network Cymru, it is estimated that 730 patients from 3 participating centres can be randomised to folic acid 5 mg daily or placebo over 1 to 2 years. We estimate that 5% will present with B12 deficiency and 10% with folate deficiency at baseline. These patients will not enter the main trial but will be treated appropriately and followed-up as part of the comprehensive cohort. Although up to 33% of patients with depressive illness have decreased plasma and red cell folate levels [8], not all will be considered folate deficient.

After allowing for 10% loss at each follow-up, and the exclusion of those with B12 and folate deficiency, a total of 730 patients recruited would yield 400 participants completing the trial. We estimate that this would yield 80% power, when using a 5% significance level, to detect a difference between the folic acid and placebo groups, of 3 points on the Beck Depression Inventory (BDI), generally considered a meaningful difference (equivalent to an effect size of 0.3).

Analysis will be by intention to treat. We shall compare folic acid and placebo using analysis of covariance to take account of baseline differences, notably in folate levels. We shall use multi-level modelling to test for heterogeneity across sites, and general linear modelling to assess relative contributions to differences in clinical outcomes. These include stratification variables (sex, new or continuing patient, and type of anti-depressant); biochemical variables (especially baseline folate, functional folate deficiency, homocysteine and methylation status); demographic variables [especially smoking, alcohol and drug consumption – all known to affect homocysteine levels [38]]; genetic variables (including interactions with biochemical variables); and pharmacological variables. Before modelling we shall develop an explicit analysis plan exploiting previous studies, for example the finding that baseline folate predicts response to antidepressant better than homocysteine does [39].

Our pharmaco-economic analysis will evaluate folate augmentation through cost-utility analysis and estimated cost-effectiveness ratios. Post hoc analysis will also estimate the cost-utility of pharmacogenetic testing, if results suggest this has predictive value. We shall prospectively collect major direct costs to the NHS of the health care resources used by participants. These will include investigations, treatments, use of primary and secondary care services, trial drug therapy, management of adverse drug reactions and genotyping. To estimate cost-effectiveness ratios, we shall estimate treatment effectiveness through utility scores derived from the EQ-5D. We shall take account of uncertainty by 'bootstrapping'. We shall identify potential predictors of response through regression analysis, and thus analyse the cost-effectiveness of genetic and biochemical testing post hoc. We shall compare the estimated incremental cost per QALY of folate augmentation for new and continuing antidepressant users, and of laboratory testing, with the results of other economic assessments of antidepressant treatments.

### Economic analysis

We will assess whether the use of folic acid supplementation is cost-effective by estimating the incremental cost-utility and cost-effectiveness ratios of antidepressant drug plus folic acid, relative to antidepressant drug alone. Further, a post-hoc analysis will be conducted to estimate the cost-utility and cost-effectiveness of pharmacogenetic testing, should the trial results suggest that genetic testing is of predictive value.

#### Healthcare resource utilisation

We shall use prospective self-complete questionnaires to assess patients' use of health and social services. These will be collected by the research professional at baseline, week 4 and week 12 of treatment. The questionnaire will include items on patients' use of general practice and community nursing services and social services. We shall supplement collection of healthcare resource utilisation data by reviewing patients' GP records after the final 12-week follow-up. This will allow for the collection of data on hospital clinic attendance, inpatient admission and home visits etc.

#### Cost analysis

The perspective of the NHS will be adopted with the major direct costs of health care resources used by patients in the trial being collected prospectively. These will include treatments and investigations, use of primary and secondary care clinic services, concomitant and trial drug therapy, management of adverse drug reactions and genotyping. Unit costs will be sought from appropriate sources [27,40,41].

#### Cost-utility analysis

For the purposes of estimating the cost-utility ratio, treatment effectiveness will be assessed by eliciting utility scores from trial participants. Patients will be asked to complete the EQ-5D questionnaire and Visual Analogue Scale at the time points specified. We shall also conduct a cost-effectiveness analysis by considering the incremental cost per depression-free week. The number of weeks free from depression will be calculated from analysis of the depression symptom rating scales, and by assuming linear interpolation between time points.

#### Uncertainty analysis

Sensitivity analyses will be conducted to test the robustness of our findings. We shall use such analyses based on the observed distributions of outcome and costs to test whether, and to what extent, the incremental cost-utility and cost-effectiveness ratios are sensitive to key assumptions in the analysis. Uncertainty will also be addressed by means of probabilistic sensitivity analysis with results presented as cost-effectiveness acceptability curves.

#### Subgroup analysis

Putative predictors of response, adverse reactions and high cost episodes will be tested by means of generalised linear regression models. These will be used to inform the post-hoc analysis of the cost-effectiveness of pharmacogenetic testing.

#### Generalisability and policy implications

The findings of the economic evaluation will be compared with the results of other health economic assessments of antidepressant drug treatments, including the AHEAD trial, a randomised control trial to compare the cost-effectiveness of tricyclic antidepressants, selective serotonin re-uptake inhibitors and lofepramine, funded by the HTA programme [42]. The estimated cost per QALY (and cost per depression-free week) of folate supplementation, and of pharmocogenetic testing, will be compared with other health care interventions to place into context the value for money they may offer.

### Direct access to source data/documents

Trial related monitoring, audits, Research Ethics Committee reviews and regulatory inspections will be permitted, allowing access to data and documents where required.

### Quality control and quality assurance

The conduct of this trial will follow the principles of good clinical practice outlined by the ICH-GCP and will comply with the EU directive 2001/20/EC. The research is underpinned by the MRC guidelines for clinical trials [43-46] and the Research Governance Frameworks for England and Wales [47,48].

A Trial Steering Committee (TSC) will be established to oversee the running of the trial, and will meet annually, with the first meeting being in Month 3. A Data Monitoring and Ethics sub-Committee (DMEC) will also be established. This committee will meet independently of the TSC but will be responsible for reporting to it.

Two other related work groups will be established to manage the project, a Trial Management Group (TMG) and a Research Team (RT). The lead applicant will chair the TMG which will meet every three months and consist of co-applicants, collaborators, the trial co-ordinator and service user and public involvement representatives. The TMG will report to the TSC, and oversee the work of the RT. The RT, chaired by the trial co-ordinator and consisting of the three research professionals, will be responsible for the day-to-day research activities. The RT will meet and obtain input from particular members of the TMG when relevant. Members of the TMG will also have the opportunity to comment on draft questionnaires, draft papers and any other trial material. The existing close working relationship between members of the proposed TMG will ensure the aims and objectives of the project will be met within the time specified.

Full ethical approval has been sought from the Multi-centre Research Ethics Committee (MREC) for Wales and from the Local Research Ethics Committees (LRECs) at each centre. An Eudract Number (2006-004647-37) and clinical trial authorisation (CTA) have been received via the Medicines and Healthcare products Regulatory Authority (MHRA). We have ensured that there is appropriate insurance/indemnity to cover the liability of the investigators. In addition, we will ensure that we obtain written informed consent from all patients entering into the trial and monitor, record and report any serious unexpected adverse reactions to the TSC and DMEC, the sponsor, the MHRA and ethics committees as appropriate. An annual safety report will be provided.

### Data Monitoring and Ethics Committee (DMEC)

The role of the DMEC is to consider the need for interim analyses of trial data, the implications of such analyses, and requests for release of interim data. It will report to the TSC about these issues and after each meeting. As this is a health technology assessment rather than a pharmaceutical trial it is less likely that the DMEC will require interim analysis of trial data.

If two or more serious adverse events (SAEs) occur in either of the folic acid and placebo groups, the research team will report this to the chair of the DMEC. The DMEC will examine the evidence, and if there is evidence of imbalance in SAEs between the treatment groups that requires further action, report this to the TSC.

If new evidence becomes available during the course of the trial, for example suggesting that folic acid is substantially better or worse than no supplement, it is the responsibility of the DMEC to consider such issues and make recommendations on the continuation of the trial to the TSC.

### Ethics

MREC for Wales and appropriate LREC approval have been sought. All trial documentation, including patient information leaflet and consent form, referral forms and template GP letters have been submitted for approval. To conform to the data protection and freedom of information acts, all data will be anonymised and stored securely. No published material will contain patient identifying information.

### Obtaining informed consent from participants

Only patients 18 years of age or over and giving informed consent will participate in the trial. Informed consent will be obtained during the screening interview where both a psychiatrist and research professional will be present to ensure that the patient fully understands the nature of the trial and answer any questions. Patients will also be informed that they can withdraw from the trial at any point and that doing so would not affect the care they received. Patients will be given a copy of their consent form to keep.

Patients unable to give informed consent will be excluded from the trial.

### Ethical issues of DNA testing

All genetic studies to be undertaken in the trial will be subject to approval by a research ethics committee accredited to deal with multi-domain studies. It is also important to note that the studies will be conducted under a strict ethical framework that adheres to guidelines developed by Department of Health, MRC and the Nuffield Council on Bioethics. The studies will also be in accordance with the Human Tissue Act [49]. The following are the key points of the study to be undertaken, as raised by the reviewers:

• The DNA samples will be transported from the site of patient recruitment to Liverpool in a coded form where they will be extracted and stored. The laboratory will only know the samples by code numbers, and will not be told of clinical details including the study arm into which the patient has been randomised, until after all the genotyping has been completed, and the results analysed. Therefore all genotyping will be blinded.

• The DNA will be stored in a coded form until the end of the study. When the DNA has been linked to the anonymised clinical details, the DNA sample will be irreversibly anonymised and the original code destroyed.

• All patients will be asked to take part in the genetic study. In other genetic studies being undertaken in Liverpool, no patient has yet refused on the basis of the fact that DNA was being collected. Furthermore the refusal rate in studies involving a single blood sample for DNA analysis is <1%. However, as the trial progresses, we will continue to monitor the situation, and if there are problems associated with recruitment which can be related to the genetic testing, then steps will be undertaken to rectify the situation after consultation with the ethics committee.

All patients will be asked to give informed consent for the genetic study. Thus additional consent, separate from consenting to the main study, will be required. Participants will be asked to give specific consent to allow us to use the participant's DNA samples to investigate the specific genes mentioned in the protocol and broad consent to allow us to use their anonymised samples for further genetic testing. Anonymised DNA samples will be used in the future to look at other genes relevant to responsiveness to folate, as and when these are identified. Participants who consent to the specific genetic testing will be invited to give broad additional consent to anonymise their DNA samples for analysis conducted once the FolATED trial is complete. Where no additional consent is given, the DNA sample will be destroyed at the end of the study. The patient information leaflet will state that these DNA samples will be considered to be a gift and will be stored under the custodianship of University of Liverpool. No information on the genetic analysis will be passed onto the patients individually or to their GPs.

### Data handling and record keeping

Patient information will only be accessible to the research team. All data will be link anonymised so that no patient identifying information will be kept with raw data. All files will be kept with the local research teams in a locked and secure cabinet. It is our intention however, to attempt to make this trial as paperless as possible, thus most of the data will be recorded electronically. Electronic data will be stored on a central computer at each centre. Field investigators will use laptops that will be cleared of data after every visit once uploaded to the central database. The database will be designed to ensure only valid data can be entered. Anonymised data will be collated by NWORTH centrally from the three participating sites on a regular basis for ongoing analysis and quality assurance monitoring. Since NWORTH will also be coordinating the randomisation procedures, the randomisation codes will be kept on a separate database by NWORTH.

With regard to blood samples, they will be split for different analyses depending on the consent given by the participant. The first sample will be sent to the local pathology laboratory for routine haematology analysis (FBC, red cell folate, serum folate and B12), the samples will then be destroyed. A second sample will be sent to Cardiff for analysis of homocysteine levels, these samples will also be destroyed following analysis. A third sample, only taken with additional consent, will be used for DNA analysis at the University of Liverpool and will be viewed as a gift to the University. The samples will be stored at the Genetic laboratory within the University with no identifying information for the duration of the study. Blood samples used for genetic analyses and homocysteine will be coded so that no patient identifying information will be available with the sample. Blood samples used for routine haematology will have patient details for clinical reasons so that results can be forwarded to appropriate GPs and to detect folate deficiency during the course of the trial. To safeguard the research team's blindness to treatment, a collaborating GP will monitor blood results for changes in folate levels during the trial. We shall keep an extra sample of the plasma at each research site as a back up for homocysteine analysis. This is to safeguard against loss of samples through unforeseen events like equipment failure. We shall destroy these stored samples at the end of the study.

Anonymised electronic data will be stored by NWORTH following the trial completion. This allows future access to raw data. Consent forms will be stored securely at local sites according to local research governance procedures. All blood samples retained for genetic analysis will be destroyed after five years unless participants give broad consent to allow anonymised samples to be kept indefinitely for future analysis. The database linking subject identity to anonymisation codes will be stored securely at each local site for a period of five years following the completion of the trial. This database will then be destroyed, ensuring trial data are permanently and irreversibly anonymised.

## Discussion

### Finance

The FolATED trial is funded by a grant from the NHS Health Technology Assessment Programme to Bangor University. No drug manufacturing company is sponsoring the trial. Folic acid manufactures have had no involvement with the design nor will they be involved with the management or reporting of the trial. DHP Ltd are manufacturing the study drugs folic acid 5 mg and matching placebo specifically for the study on a commercial basis. This ensures that adequate objectivity with regard to the study findings.

### Cost implications

FolATED has been designed to minimise costs for participating hospitals, GPs and Community Mental Health Teams (CMHTs). The drugs are supplied to patients free-of-charge. Folic acid is a simple out-patient treatment and the follow-ups scheduled for the study are only marginally more than those that would be required with standard antidepressants treatment outside the trial. To reimburse the administration costs for GPs to refer patients to the trial a payment of £50 will be made to GPs for each patient recruited into the trial.

### Indemnity

No special arrangements have been made for compensation for non-negligent harm suffered by patients as a result of participating in the study. Folic acid 5 mg is licensed for use as a treatment for folate deficiency. A lower dose, folic acid 400 mcg is available over-the-counter as a vitamin supplement and often used by pregnant women. The NHS indemnity liability arrangements will apply for negligence on the part of any healthcare professional involved in the study.

### Publication policy

A detailed publication strategy will be devised once the trial has started. We are committed to publishing in as wide afield as possible in peer reviewed journals and to ensuring that appropriate recognition is given to anyone who has worked on trial. We are also committed to making research finding accessible for secondary analysis.

## Conclusion

If folic acid is shown to improve the efficacy of antidepressants, then it will provide a safe, simple and cheap way of improving the treatment of depression in primary and secondary care.

## Abbreviations

AE: Adverse Event;

AR: Adverse Reactions;

BDI: Beck Depression Inventory;

BNF: British National Formulary;

BU: Bangor University;

CGI: Clinical Global Impression;

CMHTs: Community Mental Health Teams;

CTA: Clinical Trial Authorisation;

DMEC: Data Monitoring and Ethic Committee;

EDTA: Ethylenediaminetetraacetic acid;

EQ-5D: EuroQol scale;

EU: European Union;

FBC: Full Blood Count;

FDA: Food and Drug Agency;

GP: General Practitioners;

HADS: Hospital Anxiety and Depression Scale;

Hcy: Homocysteine;

ICD-10: International Statistical Classification of Diseases and Related Health Problems 10th RevisionICH-GCP – Good Clinical Practice;

LREC: Local Research Ethics Committee;

MADRS: Montomergy-Asberg Depression Rating Scale;

MHRA: Medicines and Healthcare product Regulatory Agency;

MHRN-C: Mental Health Research Network Cymru;

MRC: Medical Research Council;

MREC: Mulitcentre Research Ethics Committee;

MTHFR: Methylenetetrahydrofolate reductase:

NA: Noradrenaline;

NEW: North East Wales;

NHS: National Health Service;

NICE: National Institute of Clinical Exellence;

NWORTH: North Wales Organisation for Randomised Trials in Health;

NWW: North West Wales;

OR: Odds Ratio;

PHQ9: Patient Health Questionnaire 9;

QALY: Quality Adjusted Life Years;

RT: Research Team;

SAE: Serious Adverse Events;

SAR: Serious Adverse Reactions;

SNRI: Selective Noradrenaline Reuptake Inhibitor;

SNP: Single Nucleotide Polymorphisms;

SSRI: Selective Serotonin Reuptake Inhibitor;

SUSAR: Suspected Unexpected Serious Adverse Reaction;

TCA: Tricyclic Antidepressants;

TMG: Trial Management Group;

TSC: Trial Steering Committee;

UWB: University of Wales Bangor;

UKU: Udvalg for Kliniske Undersogelser (UKU side effect rating scale).

## Competing interests

The author(s) declare that they have no competing interests.

## Authors' contributions

ITR contributed methodological and health services research expertise and was responsible for the first study protocol leading to funding. SHR was responsible for the second and subsequent versions of the protocol, as well as coordinating and contributing to the development of the study design and protocol. All other named authors contributed to the study design and protocol in complementary ways: KL and RT contributed clinical and health services research expertise; EB and GS contributed trial management expertise; DH contributed the health economics component; MP contributed the genetics component; SM contributed expertise in the biochemistry of folate and homocysteine; RW contributed methodological and statistical expertise; and CW contributed primary care research expertise. All authors have seen and agreed this protocol manuscript.

## Pre-publication history

The pre-publication history for this paper can be accessed here:


