# Correction: Folate Augmentation of Treatment – Evaluation for Depression (FolATED): protocol of a randomised controlled trial

**DOI:** 10.1186/1471-244X-9-14

**Published:** 2009-04-07

**Authors:** Seren Haf Roberts, Emma Bedson, Dyfrig A Hughes, Keith R Lloyd, David B Menkes, Stuart J Moat, Munir Pirmohamed, Gary P Slegg, Johannes Thome, Richard Tranter, Rhiannon Whitaker, Clare Wilkinson, Ian T Russell

**Affiliations:** 1 North Wales Section of Psychological Medicine, Institute of Medical and Social Care Research (IMSCaR), Bangor University, Academic Unit, Wrexham Technology Park, Wrexham, LL13 7YP, UK; 2 Centre for Economics and Policy in Health, IMSCaR, Bangor University, Dean Street, Bangor, Gwynedd, LL57 1UT, UK; 3 CHIRAL, Institute of Life Science, School Of Medicine, Swansea University, Swansea, SA2 8PP, UK; 4 Waikato Clinical School, University of Auckland, Private Bag 3200, Hamilton 3240, New Zealand; 5 Department of Medical Biochemistry and Immunology, University Hospital of Wales, Health Park, Cardiff, CF14 4XW, UK; 6 Department of Pharmacology and Therapeutics, University of Liverpool, Ashton Street, Liverpool, L69 3GE, UK; 7 Neuroscience and Molecular Psychiatry, Institute of Life Science, School Of Medicine, Swansea University, Swansea, SA2 8PP, UK; 8 North West Wales NHS Trust, Ysbyty Gwynedd, Bangor, Gwynedd, LL57 2PW, UK; 9 North Wales Organisation for Randomised Trials in Health (NWORTH), Institute of Medical and Social Care Research (IMSCaR), Bangor University, Ardudwy Hall, Normal Site, Bangor, Gwynedd, LL57 2PX, UK; 10 Department of Primary Care and Public Health, Cardiff University, Gwenfro Building, Wrexham Technology Park, Wrexham, LL13 7YP, UK

## Abstract

This correction reports changes in our protocol since its publication. These include changes to authorship and acknowledgements, together with improvements to study design and procedures, and correction of an internal inconsistency. The improvements relate to the exclusion criteria, assessments carried out at screening, and mode of data collection.

## Correction

Publication of our protocol [1] revealed that David Menkes and Johannes Thome, whose contribution was originally cited under the Acknowledgements section, had both met the authorship criteria for this journal. So the full list of authors is revised as follows:

Seren Haf Roberts, Emma Bedson, Dyfrig A Hughes, Keith R Lloyd, David B Menkes, Stuart J Moat, Munir Pirmohamed, Gary P Slegg, Johannes Thome, Richard Tranter, Rhiannon Whitaker, Clare Wilkinson, Ian T Russell

The authors' contributions should read:

"ITR contributed methodological and health services research expertise and was responsible for the first study protocol in collaboration with GS. SHR was responsible for the second and subsequent versions of the protocol, as well as coordinating and contributing to the development of the study design. All other named authors contributed to the study design and protocol in complementary ways: KL, DM, JT, and RT contributed clinical and health services research expertise; EB and GS contributed trial management expertise; DH contributed the health economics component; MP contributed the genetics component; SM contributed expertise in the biochemistry of folate and homocysteine; RW contributed methodological and statistical expertise; and CW contributed primary care research expertise. All authors have seen and agreed this protocol manuscript."

The acknowledgments should read:

"The development of this protocol has benefited from support from: (1) three participating NHS Trusts (North East Wales, North West Wales and Swansea), in particular their Research and Development Departments, haematology laboratories and pharmacies; (2) collaborating GPs; (3) Dr Alan Woodall, who helped to develop the original hypothesis; (4) Dr Lindy Miller.

This project is funded by the NHS R&D Programme Health Technology Assessment (HTA) Programme (project number 04/35/08). The views and opinions expressed are those of the authors and do not necessarily reflect those of the Department of Health.

Since publication we have also enhanced the study protocol in several ways:

1) We have updated the exclusion criteria by replacing 'taking lithium' by bipolar disorder. This ensures that only patients with unipolar depression enter the trial, whether taking lithium or not. We have found no evidence to suggest a risk of adverse interactions between lithium and folate or other contraindications. Nevertheless this exclusion criterion was impeding recruitment into the trial, particularly from secondary care. Because the summary of product characteristics states that "folate supplements enhance the efficacy of lithium therapy", we shall account for this through analysis of covariance. We have amended the Statistics section to reflect this. The second sentence of the third paragraph now reads "We shall compare folic acid and placebo using analysis of covariance to take account of baseline differences, notably in folate levels *and the prescription of lithium*."

2) We have removed from the screening appointment the majority of assessments including the MADRS, CGI, EQ5D, SF12, and UKU. Patients still complete the BDI, which is the primary outcome measure. This has helped to reduce the burden on the patient and has increased the number of patients who can be screened by psychiatrists. The full battery of assessments at randomisation, which provides the true baseline, continues as before. To reflect this change we have removed the first sentence from the section on baseline measures and blood samples. Table 1 also incorporates this change.

3) Though we had intended the FolATED trial to be paperless, we have replaced the electronic trial database by a rigorous paper-based data collection system. We have therefore amended the section on data handling and record keeping, and removed the following sentences: "It is our intention however, to attempt to make this trial as paperless as possible; thus most of the data will be recorded electronically. Electronic data will be stored on a central computer at each centre. Field investigators will use laptops that will be cleared of data after every visit once uploaded to the central database. The database will be designed to ensure only valid data can be entered."

4) There was an inconsistency between timing of the questionnaire administrations quoted in the health resource utilisation section and Table 1 of the protocol. The questionnaires are administered at second baseline, 12 weeks and 6 months rather than baseline, 4 week and 12 weeks. The second sentence of the section on healthcare resource utilisation now reads "These will be collected by the research professional at the *second baseline*, week *12 *and month *6."*

## Pre-publication history

The pre-publication history for this paper can be accessed here:


